# ‘Role Model Moments’ and ‘Troll Model Moments’ in Surgical Residency: How Do They Influence Professional Identity Formation?

**DOI:** 10.5334/pme.1262

**Published:** 2024-05-20

**Authors:** Jeroen Bransen, Martijn Poeze, Marianne C. Mak-van der Vossen, Karen D. Könings, Walther N. K. A. van Mook

**Affiliations:** 1 Department of Trauma Surgery, Maastricht University Medical Center+, The Netherlands; 2 School of Health Professions Educations, Maastricht University, The Netherlands; 3 Assistant professor in medical education, Amsterdam UMC, Department of General Practice, Amsterdam, The Netherlands; 4 Amsterdam Public Health research institute, Amsterdam, The Netherlands; 5 School of Health Sciences, University of East Anglia, UK; 6 Department of Intensive Care Medicine, and postgraduate dean, Academy for Postgraduate Training, Maastricht University Medical Center+, Maastricht, The Netherlands

## Abstract

**Introduction:**

Role models are powerful contributors to residents’ professional identity formation (PIF) by exhibiting the values and attributes of the community. While substantial knowledge on different attributes of role models exists, little is known about their influence on residents’ PIF. The aim of this study was to explore surgical residents’ experiences with role models and to understand how these contribute to residents’ PIF.

**Methods:**

Adopting a social constructivist paradigm, the authors used a grounded theory approach to develop an explanatory model for residents’ experiences with role models regarding PIF. Fourteen surgical residents participated in individual interviews. The authors iteratively performed data collection and analysis, and applied constant comparison to identify relevant themes.

**Results:**

Role model behavior is highly situation dependent. Therefore, residents learn through specific ‘role model moments’. These moments arise when residents (1) feel positive about a moment, e.g. “inspiration”, (2) have a sense of involvement, and (3) identify with their role model. Negative role model moments (‘troll model moments’) are dominated by negative emotions and residents reject the modeled behavior. Residents learn through observation, reflection and adapting modeled behavior. As a result, residents negotiate their values, strengthen attributes, and learn to make choices on the individual path of becoming a surgeon.

**Discussion:**

The authors suggest a nuance in the discussion on role modelling: from ‘learning from role models’ to ‘learning from role model moments’. It is expected that residents’ PIF will benefit from this approach since contextual factors and individual needs are emphasized. Residents need to develop antennae for both role model moments and troll model moments and acquire the skills to learn from them. Role model moments and troll model moments are strong catalysts of PIF as residents follow in the footsteps of their role models, yet learn to go their own way.

## Introduction

Professional identity formation (PIF) in residency training is defined as a learning process in which individuals learn to think, act and feel like a medical specialist [1]. When young trainees transform into fully skilled specialists, new professional identities are socially constructed, resulting from an integration of personal identity and the professional standards of the community [2,3,4,5,6]. An essential goal of residency training is, thus, to support residents in developing a professional identity. Role models can be powerful contributors to residents’ PIF, because they exhibit the attributes, behaviors, and norms and values of the community [7,8]. However, it is unclear whether learners actually adopt the attributes they say they admire [9,10]. Differentiating between desirable and undesirable behavior can be a complex task, and there is a risk of unintentionally emulating unfavorable conduct [9,11]. The repercussions of professionalism lapses are widely recognized [12,13,14,15,16,17,18,19,20]. Although it is beyond debate that learning from role models is a strong teaching and learning strategy in medical education [7,21,22,23,24,25,26], the relation between residents’ PIF and role modelling is not fully understood. While substantial knowledge on different attributes of role models exists [9,21,27,28,29,30,31,32], it is unclear which attributes residents exactly emulate, and how they do so. To effectively support residents in developing a professional identity, it is, therefore, essential to bridge the gap in our understanding of how residents adopt behaviors, attributes, norms, and values from their role models.

Role models are described as excellent clinicians who are displaying a humanistic style of teaching, and demonstrating a high degree of professionalism, while negative role models are described as being uncaring towards patients, and unsupportive towards learners [27]. The three educational levels that role models function in are: the formal, the informal, and the hidden curriculum [33]. Positive role modelling happens on all three levels, while negative role modelling happens mainly in the informal and hidden curriculum [17,34]. In the formal curriculum, role modelling can be used as a learning process through observing the intentional actions of role models, followed by reflection [25,35]. However, learning from role models in the informal and hidden curriculum can be challenging, considered the strong and implicit nature of learning within these contexts. Thus, if we keep trying to improve role modelling by focusing on the intentional positive behavior of role models, we will continue to underestimate the influence of the informal and hidden curriculum on residents’ perceptions.

Drawing from Bandura’s social learning theory [36], researchers have proposed that residents learn from their role models by ‘observation and apperception’ [27], or by ‘observation, reflection, and reinforcement’ [25]. In their studies among medical students and medical specialists, Passi and colleagues found that learning by positive role modelling occurs through observation and judgement, followed by trying out the modelled behavior [24,34]. However, learning contexts for students and residents differ, as do their stages of personal and professional development.

The theory of Communities of Practice (CoP) provides a useful scaffolding framework for research related to PIF, emphasizing the social nature of learning [37,38,39]. Residents develop their professional identity within the CoP through a process of participation and socialization [8,40,41]. In the beginning of their training, residents find themselves in the periphery of the community. Their participation is legitimate because they were admitted to the training program. Over time, they move from legitimate peripheral participation to full participation, internalizing the norms and values of the community and thereby developing the identity of community members. Although residents are constantly interacting with many members of the CoP, not every member is an ‘individual admired for their ways of being and acting as professionals’ [26] in every situation for every single resident. Such an excellent clinician would be considered a unicorn. Since a person’s behavior may be influenced by the situation [42], we assume that contextual factors play an important role on both the learning process and the learning results. Similar experiences might lead to different learning outcomes, depending on personal characteristics and needs of those entering the CoP. The theoretical framework of CoP allows us to focus on both the social process of identity formation and on resident’s individual journeys.

The development of a professional identity stands as a key objective in residency training. To effectively support residents on this journey, it is crucial to understand how residents are shaped by the interactions with their role models throughout this process. This study aims to answer the research question: how is PIF influenced by role modelling, from the perspective of residents?

## Methods

### Study design

We adopted a social constructivist paradigm and used an approach based on constructivist grounded theory (CGT) [43]. We considered CGT to be an appropriate methodology since our goal was to develop an explanatory model for residents’ experiences with role models. For the inquiry into this social phenomenon, we conducted individual online interviews with surgical residents. We expected that the amount of experiences with role models would be abundant, because of the intensive guidance by supervisors when engaged in different activities (e.g. meetings and patient activities in the clinic, emergency department, operating room) and the many possibilities to observe supervisors’ behavior.

### Research team and reflexivity

Within our qualitative methodological design, meaning is constructed through interactions of researchers with participants. Background information of the researchers is thus relevant to acknowledge. JB and MP are staff trauma surgeons at Maastricht University Medical Center (Maastricht UMC+) and have experienced the process of PIF within this context. They are both members of the surgical training group. MM has been trained as a general practitioner, and is now a medical educator and medical education researcher at the training program for General Practitioners at Amsterdam University, focusing on professionalism and learning in the workplace. KK is a cognitive psychologist and medical education researcher at Maastricht University School of Health Professions Education (SHE). WvM is an intensivist at Maastricht UMC+ and the dean for postgraduate medical education, with a PhD on professionalism. Each member brought an unique perspective to our team, which was valuable in designing the study, analyzing the data and reporting our results.

### Setting and context

Surgical residency in the Netherlands is organized in seven training regions, each with one university medical center (UMC) and several affiliated teaching hospitals. The Netherlands on average has 450–500 surgical residents, 48–55 of whom are trained in the region of Maastricht UMC+, in which this study was conducted. Maastricht UMC+ has four affiliated teaching hospitals. The residency program comprises six years: four years of general surgical training, followed by two years of specialization (trauma, gastrointestinal, pediatric, oncology, or vascular surgery). Residents spend two years in a UMC.

### Sampling strategy and participants

Surgical residents were recruited via e-mail and via personal contact with local program directors. An e-mail was sent to the entire eligible population. We used convenience sampling: everyone who responded was interviewed and we sent re-invites until our sample was sufficient. Before determining data sufficiency, we ensured a variety of participants in terms of years of training and current training hospital.

### Data collection

Individual interviews were chosen as the preferred instrument based on the assumption that stories on experiences and reflection contain sensitive information and that the influence of role models is highly variable between residents. Interviews were done in a semi-structured format [44]. We developed the interview guide based on the contemporary literature on PIF and role modelling (See Supplemental files). Prior to the interviews, participants were asked to think about three examples of situations in which they were influenced by a role model’s behavior. These narratives functioned as the starting point for the conversation about how participants perceive role models and how they perceive to be influenced by them. Follow-up questions within the specific context of these narratives were used, as well as stand-alone questions to gain information on how participants think about role models in general. Data collection and analysis were done iteratively and the follow-up questions in the interview guide were adapted accordingly. To ensure participants’ privacy, interviews were conducted by an experienced, independent physician-educationalist interviewer (Scheltus van Luijk) without any connection with the residents. Interviews were conducted online, digitally audio recorded and transcribed verbatim.

### Data analysis

Initially, open coding was used. JB read the first three transcripts and coded them line by line. KK, MP, and WM independently read and coded one of the first three transcripts and individually compared their codes with JB. With this strategy all authors could familiarize themselves with the data and reach consensus on the initial coding framework. JB coded the remaining transcripts and refined the coding framework through constant comparison. Axial coding was used to categorize codes and to determine how these were related. Selective coding was used to find the core category that connected all codes and captured the essence of the relationship between role models and PIF in surgical residency. The evolving coding framework was challenged and discussed during weekly individual meetings between JB and the other researchers, and during monthly meetings with the full research team. Through these meetings, we ensured that all researchers developed an adequate understanding of the resulting themes and their relationships, allowing them to collectively interpret the data and construct an explanatory model. This resulting model was grounded in the data, which defined our data sufficiency. Data analysis was supported by ATLAS.ti 9 (ATLAS.ti Scientific Software Development GmbH, Berlin, Germany). We used the COREQ guidelines for reporting our study [45].

### Ethical considerations

Participation was voluntary and it was emphasized that participation would never negatively affect residents’ training courses. Informed consent was given by all participants prior to participation. Anonymization and pseudonymization were applied. This study has been approved by the Ethics Review Committee of Maastricht University (Faculty of Health, Medicine, and Life Sciences, approval number FHML-REC/2020/86).

## Results

In this section, we describe how 14 residents (3 female, 11 male) from training year 1–6 perceived their experiences with their role models and how these contributed to the development of their professional identity. We present our findings in the following sequence. First, we introduce the main resulting theme, i.e. the concept of ‘role model moments’. Then, describe three aspects of this concept: the emergence of a role model moment, the learning process, and the learning results (i.e. PIF). It is important to note that contextual factors, emotions, and the learning process differ significantly in positive and negative experiences. Therefore, we separately elaborate on negative role model moments using a similar structure.

### Role models’ attributes and how residents identify with them

Residents explained that role models serve as powerful examples, embodying qualities that residents envision in their future selves. The following quotes are examples of how residents perceived their role models in their ways of being and how residents identified with them. “I was often asked if I was sure I would succeed [in surgical training]. She is a role model because she is both a woman and a surgeon. She shows that it is possible and that I can succeed” (P12).

“It’s just great to run the family with your partner and at the same time build a career where both of you are happy. She also gave me personal advice on how to combine that [personal and professional life]. That also strengthened me to get through the training well” (P6).

Identification with their role models was an important factor for residents in labeling them as such. They identified with them based on characteristics, achievements, career paths, work-life balance, or propagated values. Residents also mentioned admiring role models for specific attributes, which they wished to emulate: “He is above all a very good people manager who allows [others] to function very well in his or her own strength. And that’s something I’m not good at. I would really like to develop and learn that” (P11).

### ‘Role model moments’

Besides these descriptions of the characteristics of role models, residents also reported about specific encounters with them. Residents perceived the behaviors of role models, and their personal emotions and experiences with these behaviors, to be highly situation dependent. One resident observed a junior staff member in the operating room when suddenly a bleeding occurred:

“And you just noticed a lot of stress and a bit of panic: ‘Well, I need help from the professor.’ And I remember very well that when he [the professor] entered the operating room, he was incredibly calm and said: ‘Well, guys, just keep applying pressure on that bleeding. I’m going to wash up, I’ll put gloves on soon.’… And I just noticed that everyone calmed down. He was at the table and jokingly, within two minutes, he had put in a suture and it was done. But the technical story isn’t even the most important thing; what I noticed was how well he maintained that calm, even though the situation didn’t warrant it. He made everyone calmer.” (P3)

Another resident observed her supervisor speaking with the patient and the family after a “nasty complication” occurred:

“And he was incredibly polite [and] being very open about [it]: “well, this is what happened, I’m really sorry, it wasn’t intentional,” which was already very considerate of him. And what I also found very beautiful to see was that he actually ensured that he remained a sort of team with the patient and the family: so we came in here together, and we are going to solve it together now. And I found that beautiful because I had expected, you know, sometimes there is a kind of hostility” (P4)

Residents, thus, admired certain aspects of their role models, yet always in a specific context. In addition, residents explained that they were struck by the moment in which they felt positive (or negative) about the modeled behavior: “and that was a moment for me that I thought: I want to be such a doctor” (P6). Therefore, the term ‘role model moment’ seems more appropriate than the term ‘role model’ when discussing residents’ situational learning experiences.

### Emergence of a role model moment

Role model moments can arise when residents have positive emotions about that specific moment, have a sense of involvement, and identify with their role model. How residents identify with their role models has been described above. Below we will explain the other two elements.

#### Positive emotions

Positive emotions stood out in every story about a role model moment. Residents felt “admiration” or “inspiration” after experiencing such a moment. One resident observed his supervisor giving support to their patient in the recovery ward, after unsuccessful cancer surgery: “So he [supervisor] says: “I really want to be there for her right away.” … And then…he really had a conversation with her for an hour… And I thought, if I could do that later, to be so involved with my patients and make people feel good in a bad situation, that’s wonderful.” (P3)

One resident explained that when she admires her role model, she “starts to think about [the] why”. When these positive feelings are not present, “that process doesn’t even start”. Positive emotions are thus powerful triggers for learning through reflection.

#### Sense of involvement

Residents expressed that when they felt engaged, supported, or trusted by their role models, their feeling of being a part of the CoP increased. One of the participants made a mistake on the ward and expected to be reprimanded by the supervisor, “but in contrast he reacted extremely compassionate and I just had the feeling that he completely supported me” (P11). If residents were treated as equals and supervisors e.g. can be addressed by their first name, “the atmosphere is more relaxed and I dare to ask questions or share my doubts” (P1). Role models provide a safe learning environment by valuing residents as community members and giving them a sense of involvement.

### PIF process and results

Residents unanimously indicated that role models are crucial for their PIF: “All those courses et cetera, are the tools to provide safe and good care. But what kind of person you are as a doctor and how you deal with problems is what you learn from role models. The fact that it didn’t take long before I came up with a number of role models indicates that it does play an important role for me” (P12).

Just observing a role model would be too narrow a description of the learning process, because “it goes one step beyond. It does something to you as a person” (P8). Residents could be both participants or observers in a role model moment. At that particular moment, they were not necessarily fully aware of why they experienced certain emotions, or why they identified with their role model.

Residents subsequently reflected on the situation and context, on the modeled behavior, and also on their own qualities and imperfections. Reflection could take place directly after the role model moment, but could also be prompted by new challenges for which residents recalled role model moments from their memory. Our participants expressed that conscious reflection is a crucial step in transforming role model moments into meaningful experiences, thus make them maximally contributory to PIF. Residents indicated that they observe, imitate and adapt: “I don’t think you copy something 100%, but you extract elements from it that you combine with what works for you” (P1).

By interacting with role models, residents internalized their role models’ values, strengthened attributes, and learned to make their own choices on the individual path to become a surgeon. They did not only learn medical matters, but also “social aspects, your behavior in a group, and your behavior towards your colleagues” (P9). Residents reported to concentrate mainly on absorbing knowledge and skills when they were in the beginning of their training. Later, the focus of individual residents changed based on experiences and reflection. More senior residents tended to use “cherry-picking” when they gained a better understanding of their needs. PIF in relation to role model moments thus appears dynamic over time and residents become more and more aware of what is important to them, and why, and how they want to organize their lives.

### Emergence of a negative role model moment

Residents also shared their negative experiences. In these situations, residents instantly felt they could not identify with the modeled behavior. Instead, they felt “disapproval” or even “disgust”. Residents felt detached from the role model, because of differences in personal values or a lack of support. In contrast to the combination of factors in role model moments, negative emotions predominated during and after negative role model moments. A lack of support or the creation of distance fueled these feelings. The term ‘role model’ was perceived by our participants as inherently positive, whereas the term ‘negative role model’ was perceived as a contradiction in terms. Therefore, we suggest the term ‘troll model moment’ for a negative experience. This term also emphasizes that the individual is not inherently a troll, but only is perceived as one in that particular moment. Troll model moments can nevertheless powerfully shape professional identities because residents learn what they dislike. The learning process is somewhat different because rejection of the modeled behavior is on the foreground. “I never ever want to become like that” is a strong motivation to refrain from behavior that is not in line with residents’ values or beliefs. When asked about learning from troll models, one resident explained: “I think negative role models can make a deeper impression and the learning process is faster. You know much quicker what you don’t want. … Positive behavior is something you try to adopt, whereas negative behavior is not. You can be very clear about that: I don’t want this” (P7).

## Discussion

The aim of this study was to explore residents’ experiences with role models and to understand how these contribute to their PIF. We found that residents learn from their role models through specific *role and troll model moments*, and that emotions, both positive and negative, are key to the learning process. When residents are touched by the behavior of their role model, they themselves determine the value of these moments in relation to their PIF. In addition, our findings show that surgical residents want to increase their personal qualities, become excellent clinicians, and at the same time learn to balance their work and private lives. This aligns with previous studies that have described what residents look for in their role models and how they identify with them [9,21,27,28,29,30,31,32]. Other researchers have described *catalyst moments* (experiences, conversations, reflections) in medical students [46] and *formative moments* (critical incidents) in surgical residents [47]. Our study emphasizes these claims that PIF is sparked by specific moments.

### Role and troll model moments

Foster and Roberts have previously described positive and negative role models as ‘heroes and villains’ [48]. While caution is advised with negative behaviors for possible long-term unwanted effects, both positive and negative experiences can influence PIF. Our findings align with this perspective, although we emphasize the need to contextualize role model behavior at specific moments. Foster and Roberts also emphasize the influence of interactions that engender strong emotions. Our study builds on their notion by highlighting moments when these emotions occur. We introduce the term ‘troll model moment’ to emphasize negative behavior at a specific moment without categorizing the whole individual as negative. The same person may namely exhibit positive behavior in different contexts. Our study underscores that role model moments and troll model moments are distinct entities with unique contributing factors. Further analyzing these factors can enhance our understanding of residents’ PIF.

### PIF process and results

Residents learn what is to be a surgeon, but at the same time, they learn what it is to be a resident [3]. Therefore, proclaiming (specific elements of) a professional identity as the result of residency may be premature for individual residents, depending on their year of training and personal development.

We view PIF as a continuous process that even extends beyond residency. The outcomes of learning from role models can be observed in both developmental processes and behavioral changes [49]. In our findings, residents’ behavioral changes are more noticeable than the specific processes involved in developing their sense of self or sense of belonging. While our results touch upon aspects such as self-awareness, values, and role identification, they do not delve deeply into these concepts compared to other researchers [50,51]. Therefore, we use the term ‘sense of involvement’ to accurately reflect our findings regarding residents’ participating within the CoP.

We found that residents develop values, strengthen attributes and learn to make choices by observing and selectively copying behavior that “works for you”. This is in line with other studies that have described the social process of learning from role models as: observing a role model, reflecting on role model behavior, and consequently experimenting new behaviors [21,25,27,34,36]. This might, however, be a too simple and linear representation when it comes to the development of a professional identity.

Our findings add to the literature by suggesting that emotions are essential to the learning process in both positive and negative experiences. Emotions reflect the significance of a role model moment and show that the learning process is more unpredictable and less instrumental than previously described. In a schematic representation of the contributing factors to PIF, Cruess et al. assumed that learning from role models happens through unconscious acquisition and conscious reflection [8]. We believe that highlighting the role of emotions in the learning process can support both claims. Residents instantly ‘feel’ whether they approve or reject modeled behavior. At the same time, emotions may serve as triggers for learning through reflection.

Reflection has been extensively described as a powerful tool to enhance learning [52,53]. Nguyen et al. defined reflection as: ‘… the process of engaging the self in attentive, critical, exploratory and iterative interactions with one’s thoughts and actions, and their underlying conceptual frame, with a view to changing them and with a view on the change itself’ [54]. Our model also highlights this important role of reflection, although we believe this definition sets the bar high for all our residents. Personal characteristics, training year, specific experiences and contextual factors are likely important influences. The level of reflection may even differ from time to time within a single resident. This is crucial to understand if we want to develop interventions to promote PIF through reflection. Future research into reflection on this specific topic may be useful to gain insight into how individual residents can be supported in the development of their professional identity.

### Connection between our findings and the theoretical framework

Consistent with prior research [17,34], nearly all participants’ positive experiences occurred within the informal or hidden curriculum, while their negative experiences exclusively occurred within the hidden curriculum. The concept of the informal and hidden curriculum and the theory of CoP both emphasize the importance of social learning and its implicit character [33,37,40,41,55,56]. When residents participated in shared activities with community members, they perceived some of them as role models and experienced specific moments as role model moments. These findings underscore the subjective nature of learning from role models by highlighting that these moments are shaped by contextual factors and the dynamics within the community setting. Engaging in reflective imitation of role model behavior and navigating negative examples empowers residents to develop their own professional identities. This process facilitates their transition from the periphery to the center of the community.

Cruess et al. gave a schematic representation of the multiple factors that contribute to the process of socialization when professional identities are shaped [8]. Our study builds on their model by delving deeper into the connection between residents’ experiences with their role models and their PIF. We emphasize the significance of specific role and troll model moments and their impact on the development of residents’ professional identities. In the next section, we will explain how our subjectivist inductive approach lead to the construction of a conceptual model.

### The Role & Troll Model Moment – Model

**Figure 1:**
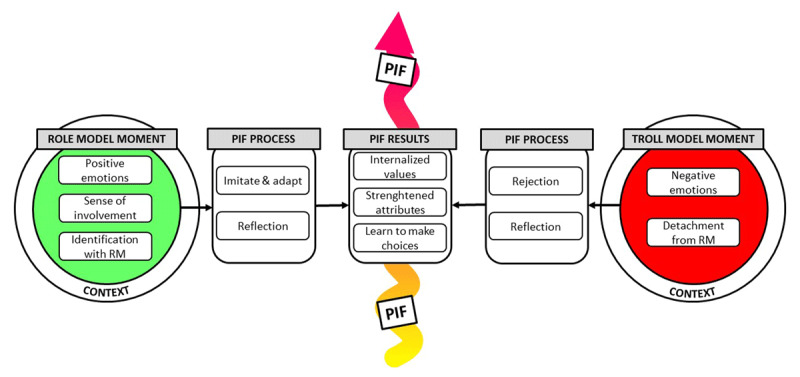
Role & Troll Model Moment-Model. Abbreviations: PIF = professional identity formation; RM = role model.

We acknowledge the complexity of PIF and the critical role of the context in which it takes place. Consequently, presenting our interpretation of the results and our comprehension of the literature in a diagram poses a challenge. While we do not imply that PIF is a linear process, we find it beneficial to use a schematic representation for clarity. Figure 1 shows the schematic representation of the relationship between role and troll model moments and PIF. A resident’s PIF is shown as a tortuous arrow, emphasizing its individual and non-linear character. As mentioned earlier, role model moments and troll model moments are not opposite ends of a spectrum. We have, nevertheless, portrayed them in this manner to clarify the different processes and to emphasize their contribution to a shared outcome, being the development of a professional identity.

### Implications

To our knowledge, this is the first time that *role and troll model moments* are described as such. Role model behavior and the way residents are being affected by it cannot be seen without a specific context.

Hence, we advocate shifting the focus from ‘learning from role models’ to ‘learning from role model moments’. This nuanced approach emphasizes the importance of the workplace context and is essential for a comprehensive understanding when discussing residents’ specific experiences.

There is limited literature on interventions enhancing PIF through role modelling in postgraduate medical education [57]. However, we believe that the necessity for interventions on role modelling per se is questionable. Our results resonate with experiential learning principles [8,58] and each component of our model offers opportunities to promote situated learning. Emphasizing the conditions for learning from role and troll model moments in daily practice can be a valuable and feasible approach.

Creating a supportive community is essential for PIF [37,38,50]. Our research reinforces this idea: when learners feel supported, engaged, and included, their sense of involvement grows, facilitating learning through role model moments. This fosters opportunities for developing a professional identity. In addition to welcoming residents and encouraging their participation, our findings suggest empowering residents to develop autonomy and make choices in their journey to becoming surgeons can further support PIF. Clinician-educators should help residents recognize both role and troll model moments. Residents then need to acquire the skills to learn from these experiences. Additionally, clinician-educators must be mindful that they are continuously serving as models themselves. Openly discussing role and troll model moments within the entire CoP can raise awareness of the value of these moments for learning among both learners and teachers. This approach allows PIF to be appreciated both on the individual psychological level and in the social context of the CoP.

### Limitations

Our study has several limitations. First, our research deliberately focused on the specific context of surgical residency in the Netherlands. While this is inherent in our methodological design, it might reduce the transferability to other contexts. The type of role model moments, and the norms and values of the surgical community may be different in other specialties, in e.g. internal medicine. However, the components of our conceptual model may not necessarily be restricted to surgical residency. We believe that PIF is comparably influenced by role model moments in any residency training and that the learning results are determined by the context of the specific experience and personal characteristics. Future research into role model moments in other specialties would be interesting to evaluate if our model could be applied to other residency training contexts.

Second, the diversity of our participant sample was limited. Three female and eleven male residents participated, and all of them were white. We were unaware of their religion and we did not explore any possible hidden cultural differences. Comparable research in other contexts with greater diversity would most likely add value to our model. For example, underrepresented residents may face cross-cultural challenges, struggle with their sense of belonging, or struggle to find a role model to identify with.

However, our goal was to conduct a first exploratory study on residents’ experiences with their role models. We conveniently sampled our participants to gain as much information on the process itself, instead of aiming for a maximum variety sample to search for individual differences.

Third, the way we asked our participants to prepare for the interview and used their narratives as a starting point for the conversation, may have unintentionally emphasized a focus on ‘moments’ from the outset. Still, the concept of the role and troll model moments only became apparent to us after several interviews and evolved throughout the course of the study. However, participants’ initial responses may have limited our perspective.

Fourth, gathering experiences from a single interview may underestimate the dynamic process of PIF over time. Previous experiences and a more matured professional identity influence the interaction between residents and their role models. In addition, different contextual factors may be important at different stages of residency training. Future studies with a longitudinal design may provide additional insights on how residents interact with their role models when their participation within the CoP increases, and how their reflection on these experiences changes over time.

Fifth, we are not aware of the exact stages of our participants’ professional identity, in terms of measuring PIF. We sampled residents from different training years, based on the assumption that we would capture residents at different stages of their PIF. Thereby we gained a valuable range of perspectives to gain an understanding of the process of PIF. However, residents in different stages of PIF may require different approaches for assistance in their development. Future studies using measurement instruments for PIF could add useful information to the discourse on PIF and learning from role model moments.

## Conclusion

We sought to understand how residents’ experiences with their role models contributed to their PIF. Through interactions with their role models and reflective practice, residents strengthened their attributes, internalized values and learned to make their own choices. Thus, interactions between residents and their role models are crucial for the development of their professional identity. Our findings add to the existing literature by asking attention for the importance of contextual factors that play an important role in this process. We propose a nuanced shift in the discourse on role modelling: from the broad concept of ‘learning from role models’ to a more focused perspective of ‘learning from role model moments’. It is crucial to recognize that role model behavior and its impact on residents cannot be fully grasped without considering the specific context in which these interactions occur. Surgical residents learn from their role models through specific *role model moments* and *troll model moments*. These moments are pivotal as residents assess their value and decide how to integrate or reject the observed behavior while shaping their professional identity. These moments act as strong catalysts of PIF as residents follow in the footsteps of their role models and at the same time learn to go their own way.

## Additional File

The additional file for this article can be found as follows:

10.5334/pme.1262.s1 Supplemental File. Interview guide.

## References

[1] Cruess RL, Cruess SR, Boudreau JD, Snell L, Steinert Y. Reframing medical education to support professional identity formation. Acad Med. 2014; 89(11): 1446–51. DOI: 10.1097/ACM.0000000000000427 25054423

[2] Chandran L, Iuli RJ, Strano-Paul L, Post SG. Developing “a Way of Being”: Deliberate Approaches to Professional Identity Formation in Medical Education. Acad Psychiatry. 2019. DOI: 10.1007/s40596-019-01048-4 30993596

[3] Jarvis-Selinger S, Pratt DD, Regehr G. Competency is not enough: integrating identity formation into the medical education discourse. Acad Med. 2012; 87(9): 1185–90. DOI: 10.1097/ACM.0b013e3182604968 22836834

[4] Lewin LO, McManamon A, Stein MTO, Chen DT. Minding the Form That Transforms: Using Kegan’s Model of Adult Development to Understand Personal and Professional Identity Formation in Medicine. Acad Med. 2019; 94(9): 1299–1304. DOI: 10.1097/ACM.0000000000002741 31460919

[5] Monrouxe LV. Identity, identification and medical education: why should we care? Med Educ. 2010; 44(1): 40–9. DOI: 10.1111/j.1365-2923.2009.03440.x 20078755

[6] Wald HS. Professional identity (trans)formation in medical education: reflection, relationship, resilience. Acad Med. 2015; 90(6): 701–6. DOI: 10.1097/ACM.0000000000000731 25881651

[7] Cruess SR, Cruess RL, Steinert Y. Role modelling—making the most of a powerful teaching strategy. BMJ. 2008; 336(7646): 718–21. DOI: 10.1136/bmj.39503.757847.BE 18369229 PMC2276302

[8] Cruess RL, Cruess SR, Boudreau JD, Snell L, Steinert Y. A schematic representation of the professional identity formation and socialization of medical students and residents: a guide for medical educators. Acad Med. 2015; 90(6): 718–25. DOI: 10.1097/ACM.0000000000000700 25785682

[9] Paice E. How important are role models in making good doctors? BMJ. 2002; 325(7366): 707–10. DOI: 10.1136/bmj.325.7366.707 12351368 PMC1124228

[10] Benbassat J. Role Modeling in Medical Education: The Importance of a Reflective Imitation. Academic Medicine. 2014; 89(4). DOI: 10.1097/ACM.0000000000000189 PMC4885588 24556777

[11] Said M, Jochemsen-Van Der Leeuw RHGA, Spek B, Brand PLP, Van Dijk N. Role modelling in the training of hospital-based medical specialists: a validation study of the Role Model Apperception Tool (RoMAT). Perspectives on Medical Education. 2019; 8(4): 237–45. DOI: 10.1007/S40037-019-00527-6 31347034 PMC6684559

[12] Rosenstein AH. Disruptive Physician Behavior Contributes to Nursing Shortage. Physician Executive. 2002; 28(6): 8. 12448134

[13] Rosenstein AH. The Quality and Economic Impact of Disruptive Behaviors on Clinical Outcomes of Patient Care. American J Med Qual. 2011; 26(5): 372–9. DOI: 10.1177/1062860611400592 21511883

[14] Rosenstein AH. Physician disruptive behaviors: Five year progress report. World Journal of Clinical Cases. 2015; 3(11): 930. DOI: 10.12998/wjcc.v3.i11.930 26601095 PMC4644894

[15] Hickson GB. Patient Complaints and Malpractice Risk. JAMA. 2002; 287(22): 2951. DOI: 10.1001/jama.287.22.2951 12052124

[16] Rawson JV, Thompson N, Sostre G, Deitte L. The cost of disruptive and unprofessional behaviors in health care. Acad Radiol. 2013; 20(9): 1074–6. DOI: 10.1016/j.acra.2013.05.009 23931419

[17] Baldwin DC, Jr., Daugherty SR, Rowley BD. Unethical and unprofessional conduct observed by residents during their first year of training. Acad Med. 1998; 73(11). DOI: 10.1097/00001888-199811000-00019 9834704

[18] Billings ME, Lazarus ME, Wenrich M, Curtis JR, Engelberg RA. The Effect of the Hidden Curriculum on Resident Burnout and Cynicism. J Grad Med Educ. 2011; 3(4): 503–10. DOI: 10.4300/JGME-D-11-00044.1 23205199 PMC3244316

[19] Hilliard R, Harrison C, Madden S. Ethical conflicts and moral distress experienced by paediatric residents during their training. Paediatrics & Child Health. 2007; 12(1): 29–35. DOI: 10.1093/pch/12.1.29 19030336 PMC2528670

[20] Rosenbaum JR, Bradley EH, Holmboe ES, Farrell MH, Krumholz HM. Sources of ethical conflict in medical housestaff training: a qualitative study. Am J Med. 2004; 116(6): 402–7. DOI: 10.1016/j.amjmed.2003.09.044 15006589

[21] Passi V, Johnson S, Peile E, Wright S, Hafferty F, Johnson N. Doctor role modelling in medical education: BEME Guide No. 27. Med Teach. 2013; 35(9): e1422–e36. DOI: 10.3109/0142159X.2013.806982 23826717

[22] Birden H, Glass N, Wilson I, Harrison M, Usherwood T, Nass D. Teaching professionalism in medical education: A Best Evidence Medical Education (BEME) systematic review. BEME Guide No. 25. Med Teac. 2013; 35(7): e1252–e66. DOI: 10.3109/0142159X.2013.789132 23829342

[23] Kenny NP, Mann KV, MacLeod H. Role Modeling in Physicians’ Professional Formation: Reconsidering an Essential but Untapped Educational Strategy. Acad Med. 2003; 78(12). DOI: 10.1097/00001888-200312000-00002 14660418

[24] Passi V, Johnson N. The impact of positive doctor role modeling. Med Teach. 2016; 38(11): 1139–45. DOI: 10.3109/0142159X.2016.1170780 27089216

[25] Park J, Woodrow SI, Reznick RK, Beales J, Macrae HM. Observation, Reflection, and Reinforcement: Surgery Faculty Members’ and Residents’ Perceptions of How They Learned Professionalism. Acad Med. 2010; 85(1): 134–9. DOI: 10.1097/ACM.0b013e3181c47b25 20042839

[26] Côté L, Leclère H. How Clinical Teachers Perceive the Doctor—Patient Relationship and Themselves as Role Models. Acad Med. 2000; 75(11). DOI: 10.1097/00001888-200011000-00020 11078674

[27] Jochemsen-van der Leeuw HGAR, van Dijk N, van Etten-Jamaludin FS, Wieringa-de Waard M. The Attributes of the Clinical Trainer as a Role Model: A Systematic Review. Acad Med. 2013; 88(1). DOI: 10.1097/ACM.0b013e318276d070 23165277

[28] Wright S. Examining what residents look for in their role models. Acad Med. 1996; 71(3). DOI: 10.1097/00001888-199603000-00024 8607931

[29] Cope A, Bezemer J, Mavroveli S, Kneebone R. What Attitudes and Values Are Incorporated Into Self as Part of Professional Identity Construction When Becoming a Surgeon? Acad Med. 2017; 92(4): 544–9. DOI: 10.1097/ACM.0000000000001454 28351068

[30] Elzubeir MA, Rizk DEE. Identifying characteristics that students, interns and residents look for in their role models. Med Educ. 2001; 35(3): 272–7. DOI: 10.1046/j.1365-2923.2001.00870.x 11260451

[31] Scott MW, Joseph AC. Excellence in role modelling: insight and perspectives from the pros. Can Med Assoc J. 2002; 167(6): 638. 12358197 PMC122026

[32] Maker VK, Curtis KD, Donnelly MB. Are you a surgical role model? Cur Surg. 2004; 61(1): 111–5. DOI: 10.1016/j.cursur.2003.09.003 14972185

[33] Hafferty FW. Beyond curriculum reform: confronting medicine’s hidden curriculum. Acad Med. 1998; 73(4). DOI: 10.1097/00001888-199804000-00013 9580717

[34] Passi V, Johnson N. The hidden process of positive doctor role modelling. Med Teach. 2016; 38(7): 700–7. DOI: 10.3109/0142159X.2015.1087482 26524562

[35] Balmer D, Serwint JR, Ruzek SB, Ludwig S, Giardino AP. Learning Behind the Scenes: Perceptions and Observations of Role Modeling in Pediatric Residents’ Continuity Experience. Ambulatory Pediatrics. 2007; 7(2): 176–81. DOI: 10.1016/j.ambp.2006.11.005 17368413

[36] Bandura A. Social foundations of thought and action: A social cognitive theory. Englewood Cliffs, NJ, US: Prentice-Hall, Inc. 1986; xiii: 617–xiii.

[37] Cruess RL, Cruess SR, Steinert Y. Medicine as a Community of Practice: Implications for Medical Education. Acad Med. 2018; 93(2): 185–91. DOI: 10.1097/ACM.0000000000001826 28746073

[38] Cruess SR, Cruess RL, Steinert Y. Supporting the development of a professional identity: General principles. Med Teach. 2019; 41(6): 641–9. DOI: 10.1080/0142159X.2018.1536260 30739517

[39] Cruess RL, Cruess SR. Professionalism, Communities of Practice, and Medicine’s Social Contract. The J Amer Board Fam Med. 2020; 33: S50–S6. DOI: 10.3122/jabfm.2020.S1.190417 32928951

[40] Lave J, Wenger E. Situated Learning: Legitimate Peripheral Participation. Cambridge: Cambridge University Press; 1991. DOI: 10.1017/CBO9780511815355

[41] Li LC, Grimshaw JM, Nielsen C, Judd M, Coyte PC, Graham ID. Evolution of Wenger’s concept of community of practice. Imp Sci. 2009; 4(1): 11. DOI: 10.1186/1748-5908-4-11 PMC2654669 19250556

[42] Ross L, Nisbett RE. The person and the situation: Perspectives of social psychology. Mcgraw-Hill Book Company. 1991; xvi: 286–xvi.

[43] Watling CJ, Lingard L. Grounded theory in medical education research: AMEE Guide No. 70. Med Teach. 2012; 34(10): 850–61. DOI: 10.3109/0142159X.2012.704439 22913519

[44] Dicicco-Bloom B, Crabtree BF. The qualitative research interview. Med Educ. 2006; 40(4): 314–21. DOI: 10.1111/j.1365-2929.2006.02418.x 16573666

[45] Tong A, Sainsbury P, Craig J. Consolidated criteria for reporting qualitative research (COREQ): a 32-item checklist for interviews and focus groups. International Journal for Quality in Health Care. 2007; 19(6): 349–57. DOI: 10.1093/intqhc/mzm042 17872937

[46] Jarvis-Selinger S, MacNeil KA, Costello GRL, Lee K, Holmes CL. Understanding Professional Identity Formation in Early Clerkship: A Novel Framework. Acad Med. 2019. DOI: 10.1097/ACM.0000000000002835 31192797

[47] Sandhu H, Foote DC, Evans J, Santosa KB, Kemp MT, Donkersloot JN, et al. “The Story I Will Never Forget”: Critical Incident Narratives in Surgical Residency. Annals Surg. 2023; 277(3). DOI: 10.1097/SLA.0000000000005219 34534986

[48] Foster K, Roberts C. The Heroic and the Villainous: a qualitative study characterising the role models that shaped senior doctors’ professional identity. BMC Med Educ. 2016; 16(1). DOI: 10.1186/s12909-016-0731-0 PMC4986406 27530252

[49] Sternszus R, Slattery NK, Cruess RL, Cate OT, Hamstra SJ, Steinert Y. Contradictions and Opportunities: Reconciling Professional Identity Formation and Competency-Based Medical Education. Perspect Med Educ. 2023; 12(1): 507–16. DOI: 10.5334/pme.1027 37954041 PMC10637293

[50] Puranitee P, Kaewpila W, Heeneman S, Van Mook WNKA, Busari JO. Promoting a sense of belonging, engagement, and collegiality to reduce burnout: a mixed methods study among undergraduate medical students in a non-Western, Asian context. BMC Med Educ. 2022; 22(1). DOI: 10.1186/s12909-022-03380-0 PMC9047274 35484548

[51] Sawatsky AP, Matchett CL, Hafferty FW, Cristancho S, Ilgen JS, Bynum WE, Varpio L. Professional identity struggle and ideology: A qualitative study of residents’ experiences. Med Educ. 2023. DOI: 10.1111/medu.15142 PMC10592531 37269251

[52] Winkel AF, Yingling S, Jones A-A, Nicholson J. Reflection as a Learning Tool in Graduate Medical Education: A Systematic Review. J Grad Med Educ. 2017; 9(4): 430–9. DOI: 10.4300/JGME-D-16-00500.1 28824754 PMC5559236

[53] Mann K, Gordon J, MacLeod A. Reflection and reflective practice in health professions education: a systematic review. Adv Health Sci Educ Theory Pract. 2009; 14(4): 595–621. DOI: 10.1007/s10459-007-9090-2 18034364

[54] Nguyen QD, Fernandez N, Karsenti T, Charlin B. What is reflection? A conceptual analysis of major definitions and a proposal of a five-component model. Med Educ. 2014; 48(12): 1176–89. DOI: 10.1111/medu.13823 25413911

[55] Buckley H, Steinert Y, Regehr G, Nimmon L. When I say … community of practice. Med Educ. 2019; 53(8): 763–5. 30859612 10.1111/medu.13823

[56] Cruess RL, Cruess SR, Steinert Y. Medicine as a Community of Practice. Acad Med. 2018; 93(2): 185–91. DOI: 10.1097/ACM.0000000000001826 28746073

[57] Mount GR, Kahlke R, Melton J, Varpio L. A Critical Review of Professional Identity Formation Interventions in Medical Education. Acad Med. 9900. 10.1097/ACM.0000000000004904 35947478

[58] Hutchinson TA, Smilovitch M. Experiential learning and reflection to support professionalism and professional identity formation. In: Cruess RL, Cruess SR, Steinert YY (eds.), Teaching Medical Professionalism: Supporting the Development of a Professional Identity. 2nd ed. Cambridge: Cambridge University Press; 2016. pp. 97–112. DOI: 10.1017/CBO9781316178485.009

