# Supplementary material for: ‘Role Model Moments’ and ‘Troll Model Moments’ in Surgical Residency: How Do They Influence Professional Identity Formation?
Source: Perspect Med Educ. 2024 May 20;13(1):313–23. doi: 10.5334/pme.1262 (PMC11122703; doi:10.5334/pme.1262)
Supplement: Supplemental File. — Interview guide. [file pme-13-1-1262-s1.pdf]

<u>Interview guide</u>

Study working title

Professional identity formation of surgical residents: the influence of role models

Research question

How is PIF influenced by role modelling, from the perspective of residents?

Instructions for the interviewer

The following questions act as a semi-structured interview guide. Question 1 refers to preparatory assignment for participants. Prior to the interview, residents are asked to think of 3 situations in which they were affected by role model behavior. These stories function as the starting point for the conversation about how participants perceive role models and how they perceive to be influenced by them. Questions 2 to 4 are used for follow-up questions within the specific context of the answers to question 1, as well as standalone questions to gain information on how participants think about role models in general. We trust you to probe for richer information.

1. Can you tell about a situation in which role model behavior affected you?
    a. Why did you pick this situation?

2. What is a role model according to you?
    a. How do you choose your role model? Based on attributes? Based on how you are affected by role model behavior?
    b. Can a role model be both positive and negative? What is the influence of the situation? Please give examples.

3. How do you form an opinion, or judgement, about the behavior of a role model?

4. How do you use the observed behavior of a role model for your own development?
    a. How do you integrate positive behavior?
    b. How do you deal with negative behavior?
    c. How does a role model influence you?
